# Long scleral tunnel technique for prevention of drainage tube-related complications during Ahmed glaucoma valve implantation

**DOI:** 10.1097/MD.0000000000035745

**Published:** 2023-10-20

**Authors:** Ying Dai, Jun-Fang Gong, Ju-Ming Zhu, Min Zhuang, Shu Zhu, Tao Sun

**Affiliations:** a Department of Ophthalmology, The Fourth Affiliated Hospital of Nantong University, The Yancheng Clinical College of Xuzhou Medical University, The First People’s Hospital of Yancheng, Yancheng, Jiangsu Province, China.

**Keywords:** intraocular pressure (IOP), long scleral tunnel technique, modified Ahmed glaucoma valve (AGV) implantation, neovascular glaucoma (NVG), postoperative complications, proliferative diabetic retinopathy (PDR)

## Abstract

To evaluate the safety and efficacy of modified Ahmed glaucoma valve (AGV) implantation (long scleral tunnel technique) in the treatment of neovascularization glaucoma (NVG). This retrospective observational case series included 23 patients (23 eyes) diagnosed with NVG secondary to proliferative diabetic retinopathy from January 2020 to June 2021. All 23 cases received anti-vascular endothelial growth factor treatment. Then, after 3 to 7 days, these cases were treated with modified AGV implantation (long scleral tunnel technique) and were followed up for at least 6 months. The best corrected visual acuity, intraocular pressure, numbers of antiglaucoma medications used, and postoperative complications were observed at 1 week and 1, 3, and 6 months after treatment. With treatment, the mean best corrected visual acuity improved significantly (*P* < .001) from 1.62 ± 0.52 logMAR preoperatively to 1.29 ± 0.36 logMAR at the 6-month follow-up. The mean postoperative intraocular pressure was significantly lower than that before modified AGV implantation during follow-up period, decreasing from 45.48 ± 7.86 mm Hg preoperatively to 14.87 ± 1.96 mm Hg at 1 week, 18.39 ± 2.25 mm Hg at 1 month, 16.61 ± 1.47 mm Hg at 3 months, and 17.48 ± 1.38 mm Hg at 6 months (F = 256.646, *P* < .001). The median number of antiglaucoma medications used by patients also significantly decreased from 3 (3–4) preoperatively to 0 (0–1) at the 6-month follow-up after surgery (Z = −4.248, *P* < .001). Postoperative complications included hyphema in 2 cases and vitreous hemorrhage in 1 case, and all 3 patients achieved satisfactory recovery with treatment. No drainage tube-related complications occurred among our patients. Long scleral tunnel technique is a safe and effective surgical treatment for NVG with fewer drainage tube-related complications.

## 1. Introduction

Neovascular glaucoma (NVG), first described and named by Weiss in 1963, is refractory and associated with a high rate of blindness.^[1,2]^ The etiology of NVG is complex, as it is usually secondary to proliferative diabetic retinopathy (PDR), ocular ischemic syndrome, or central retinal vein occlusion.^[3–5]^ The occurrence of NVG is generally related to the primary disease. In response to retinal ischemia and hypoxia, a large amount of vascular endothelial growth factor (VEGF) is produced, which promotes neovascularization and formation of neovascularization membrane in the fundus, iris, and anterior chamber angle.^[2,4,6]^ This fibrous membrane gradually blocks the anterior chamber angle, resulting in adhesion of the iris and trabecular network, closure of the anterior chamber angle, and accordingly, increased intraocular pressure (IOP). This increase in IOP then further aggravates retinal ischemia, creating a vicious cycle that ultimately results in vision loss.^[2,4,6]^ Therefore, to preserve visual function, patients with NVG must receive comprehensive treatment, including both active treatment for the primary disease and additional treatments for NVG.

Anti-VEGF treatment, antiglaucoma treatment, and panretinal laser photocoagulation (PRP) have been developed and applied in conjunction with primary disease therapies in patients with NVG.^[3,7]^ At present, commonly used antiglaucoma surgeries include trabeculectomy, cyclophotocoagulation, ciliary body freezing, and implantation of a device for aqueous humor drainage, such as the Ahmed glaucoma valve (AGV) or Ex-PRESS mini-glaucoma drainage device.^[4]^ However, NVG alone is considered a risk factor for filtering bleb failure after trabeculectomy.^[4]^ Thus, AGV implantation has become more popular due to its advantage of being less dependent on the failure of the filtering bleb.^[4]^ Although this is an obviously effective and relatively safe surgical procedure, this technique requires a lot of surgical skills, and always requires a long learning curve for surgeons.^[8]^ In recent years, glaucoma specialists have attempted to improve AGV implantation, with the goals of shortening the surgical time and reducing intraoperative and postoperative complications.^[9,10]^ In the present study, we evaluated the clinical efficacy and safety of long scleral tunnel technique for the treatment of NVG and considered the advantages and disadvantages of the modified method.

## 2. Methods

### 2.1. Patients, preoperative data, and treatment plan

This retrospective observational case series study was conducted in compliance with the Declaration of Helsinki and approved by the ethics committee of our hospital, and all included patients provided written informed consent. Cases of NVG secondary to PDR treated in our hospital from January 2020 to June 2021 were screened for inclusion according to the following criteria: Diagnosis of PDR; Neovascularization within the iris or anterior chamber angle; IOP >21 mm Hg even after adequate IOP-lowering therapy before surgery; Closure of anterior chamber angle; History of the modified AGV procedure, and; At least 6 months of postoperative follow-up data available. Patients were not included if they met any of the following exclusion criteria: Vision loss equivalent to blindness; Previous visual loss or optic nerve injury due to other reasons; or Complication of the case due to other types of glaucoma, uveitis, or serious systemic diseases. Finally, 23 patients were enrolled in this retrospective study.

**Figure 1.**
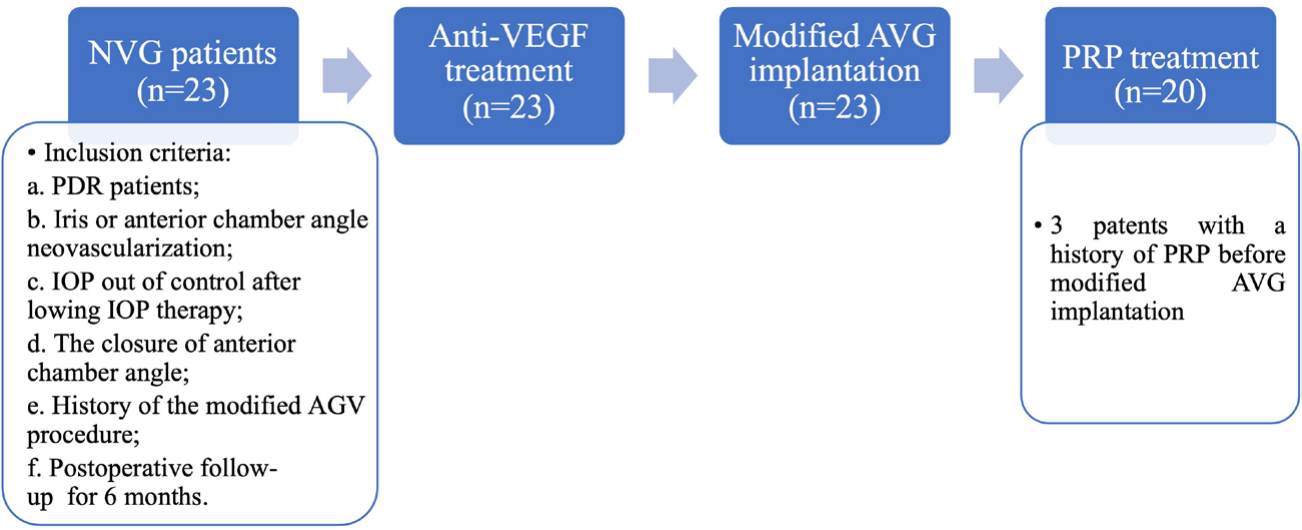
Treatment process for modified AVG implantation in NVG patients. According to the inclusion criteria, a total of 23 NVG patients were enrolled in this retrospective study. First, all eyes received anti-VEGF treatment, and 3–7 days later, modified AGV implantation was performed. Within a few months after modified AGV implantation (once the patients’ IOP returned to normal), 20 eyes received PRP treatment. Three patients had a history of PRP before modified AGV implantation. Amomg the 20 eyes, one underwent PPV together with PRP, due to the postoperative complication of VH. AGV = Ahmed glaucoma valve, IOP = intraocular pressure, NVG = neovascularization glaucoma, PPV = pars plana vitrectomy, PRP = panretinal laser photocoagulation, VEGF = vascular endothelial growth factor, VH = vitreous hemorrhage.

Before surgery, all patients underwent detailed eye examinations that included measurement of best corrected visual acuity (BCVA) and IOP, ocular B ultrasound, ultrasound biomicroscopy, stereoscopy, fundus color photography, etc. The anterior segment was evaluated by gonioscopy and ultrasound biomicroscopy examination. Additional preoperative data included history of PRP and lens status. All included eyes were treated with levofloxacin eye drops for 3 days before surgery to reduce the risk of postoperative inflammation and infection. All included eyes also received anti-VEGF treatment. Then, 3 to 7 days later, modified AGV implantation was performed. PRP treatment could be completed in the months after modified AGV implantation, as the IOP reduced to normal. The full treatment plan applied in this study is illustrated in Figure 1.

### 2.2. Surgical technique

**Figure 2.**
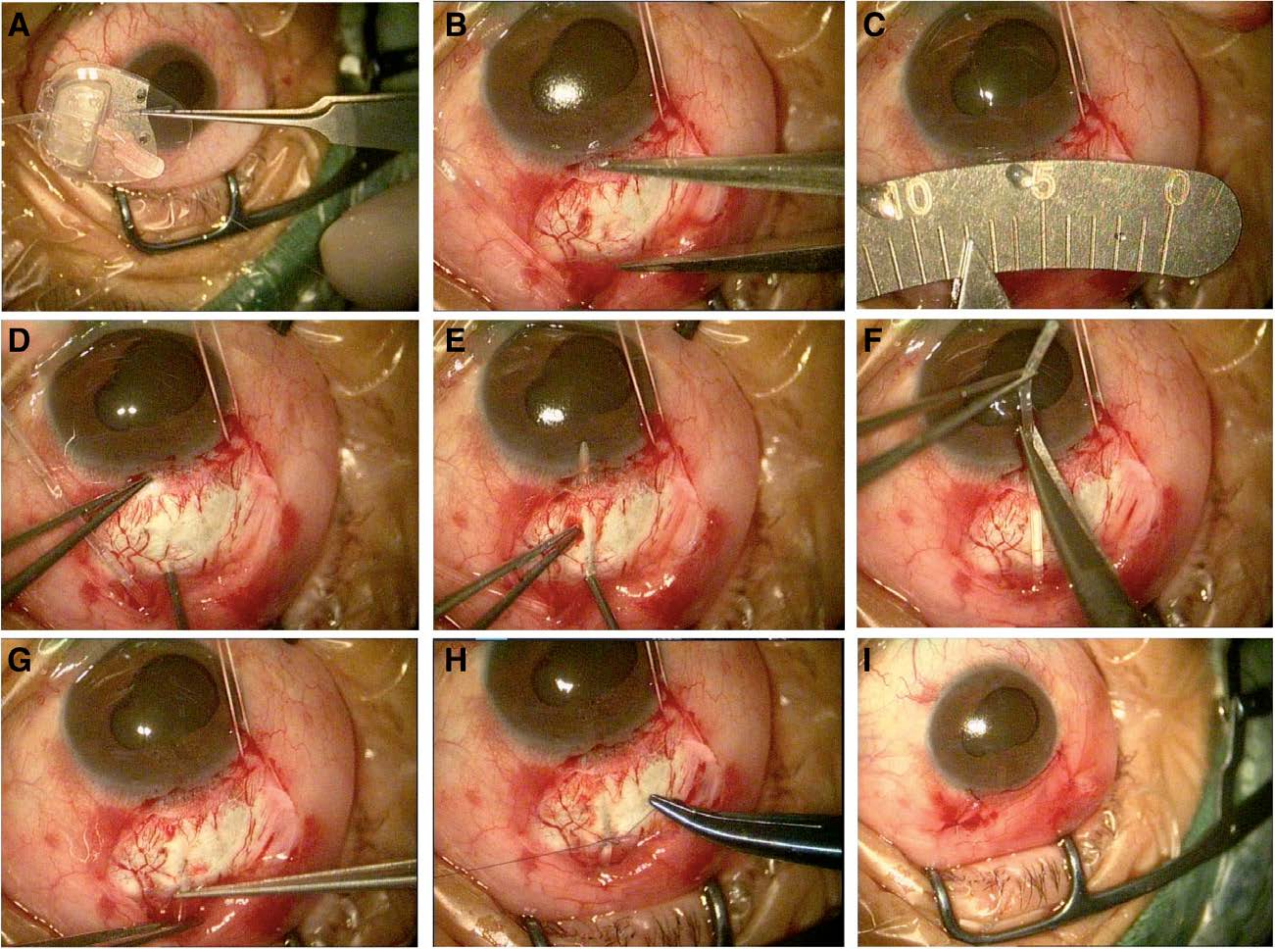
The modified operation procedure for AGV implantation for the treatment of NVG. (A) The AGV was initialized. (B–E) About 8.0 mm from the corneal limbus, a 22G syringe needle was used to puncture the corneal limbus and then enter the anterior chamber, forming a scleral tunnel about 8 mm in length at 1/2 thickness of the interlamellar sclera. (F) The tube was trimmed. (G) The tube was inserted into the scleral tunnel. (H) Sutures were placed to prevent excessive patency of the drainage tube. (I) The surgery was completed. AGV = Ahmed glaucoma valve, NVG = neovascularization glaucoma.

**Figure 3.**
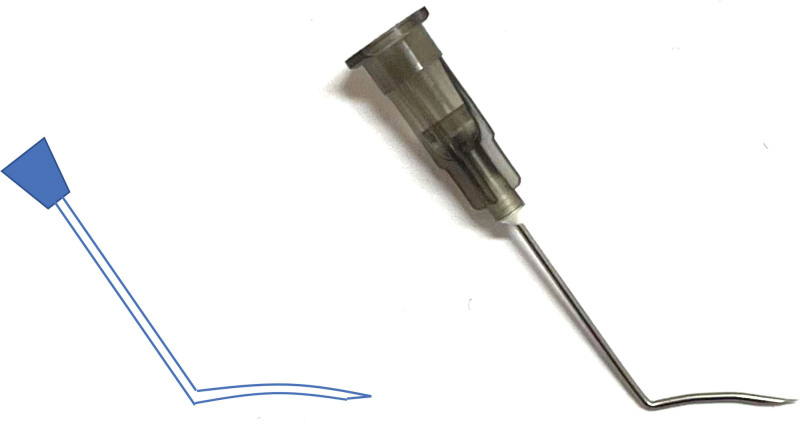
Schematic image and physical picture of the bent 22G syringe needle used during the AVG implantation surgery. An ordinary 22G syringe needle was bent at least 8 mm from the end. We recommend bending the front end of the syringe according to the radian of the eyeball, as the final bent needle will be more conducive to the operation.

The specific surgical treatment was conducted according to the following steps (Fig. 2): Parabulbar anesthesia; Cutting of the bulbar conjunctiva in the superior temporal quadrant along the corneal limbus and blunt separation along to the equator of the eyeball; Placement of 5-FU cotton tablets in the area where the drainage plate would be placed for 5 minutes, followed by rinsing with sufficient normal saline; After priming of the FP-7 Ahmed drainage valve (New World Medical, USA), suturing of the drainage plate and fixation on the sclera, leaving a clear area from the front end of the drainage plate to approximately 10 mm from the edge of the corneal limbus; and Use of a 22G syringe needle along the arc of the eyeball to create a tunnel penetration to the margin of the cornea at about 8 mm from the margin of the corneal limbus and then parallel to the iris plane into the anterior chamber, forming a scleral tunnel approximately 8 mm in length between the scleral layers. A schematic image and physical picture of the 22G syringe needle used during surgery is shown in Figure 3. Specifically, we bent an ordinary 22G syringe needle at a distance of at least 8 mm from the needle tip. We recommend bending the front end of the syringe according to the radian of the eyeball, which makes the device more conducive to the operation; In step, a tube is inserted along the tunnel without contact between the tube or the lens surface and corneal endothelium, and leaving the opening unblocked; Next, drainage tube sutures are used to prevent excessive early drainage, and; The bulbar conjunctiva is closed.

### 2.3. Postoperative assessment and outcome measures

Tobramycin and dexamethasone eye drops were administered 4 times per day for 1 week after surgery, and during the second week, patients could gradually reduce the frequency of the medication until the inflammatory response had completely subsided. The state of the cornea, IOP, drainage tube mouth, vision, fundus, and other conditions were observed regularly. Postoperative complications included shallow anterior chamber, choroidal effusion, hyphema, vitreous hemorrhage (VH), corneal endothelial decompensation, macular edema, fibrotic encapsulation around the plate, endophthalmitis, drainage tube-related complications (such as drainage tube leakage, tube erosion/exposure, and tube obstruction), etc. If postoperative complications occurred, timely symptomatic treatment was provided.

The patients were followed up for 6 to 18 months, and their visual acuity, IOP, numbers of antiglaucoma medications,and complications were observed at 1 week and then at 1, 3, and 6 months after surgery.

### 2.4. Evaluation criteria for surgical effect

Surgical success: IOP ≥ 6 mm Hg and ≤ 21 mm Hg without antiglaucoma drugs; or IOP ≥ 6 mm Hg and ≤ 21 mm Hg with antiglaucoma drugs (medication ≤ 2 types); No serious postoperative complications; and Maintenance of light perception.

Surgical failure: Inability to control IOP with medication (medication ≥ 3 types) and need for further antiglaucoma surgery; Removal of drainage valve required for any of various reasons; or Occurrence of serious postoperative complications.

Serious postoperative complications included: Drainage tube-related complications (e.g., drainage tube leakage and exposure); and Non-drainage tube-related complications (e.g., hyphemia with additional surgical treatment, VH, endophthalmitis, and others).

### 2.5. Statistical analysis

The clinical characteristics of the included patients were analyzed using IBM SPSS Statistics 25.0. Preoperative and postoperative BCVA and IOP values are expressed as mean ± standard deviation (SD). Differences between the preoperative BCVA and postoperative BCVA at 6 months were analyzed by paired *t* test. Repetitive measures analysis of variance was used for analysis of IOP at each follow-up point. Wilcoxon signed-rank test was used to analyze the difference in numbers of antiglaucoma medications before and after surgery at 6 months. When *P* < .05, the difference was considered to be statistically significant.

## 3. Results

This retrospective observational case series study included a total of 23 eyes in 23 patients, of whom 16 were male and 7 were female. The patients ranged in age from 43 to 72 years, with a mean (±SD) age of 57.47 ± 7.35 years. Among the 23 patients, 3 had a history of PRP treatment. The baseline characteristics of the included cases are summarized in Table 1.

**Table 1:** Clinical characteristics of the study population (mean ± SD).

Clinical characteristics	Total N = 23
Gender	
Male	16
Female	7
Age	57.47 ± 7.35
Preoperative logMAR VA	1.62 ± 0.52
IOP	45.48 ± 7.86
Lens status	
Phakic	21
Pseudophakic	2
Aphakic	0
Previous PRR	3

BCVA = best corrected visual acuity, IOP = intraocular pressure, PPV = pars plana vitrectomy, PRP = panretinal laser photocoagulation, SD = standard deviation.

After modified AGV implantation, the mean BCVA was significantly improved compared with the preperative BCVA, and these patients were followed up for at least 6-month. At the 6-month postoperative follow-up, the mean (±SD) preoperative BCVA showed improvement from 1.62 ± 0.52 logMAR preoperatively to 1.29 ± 0.36 logMAR (*P* < .001). Also at the 6-month postoperative follow-up, 19 of 23 eyes showed improved visual acuity, to various degrees, while visual acuity remained unchanged for 3 eyes. The 1 additional patient had decreased visual acuity.

The “hypertension” stage is very common and reaches its peak at 1 or 2 months after AGV implantation.^[8]^ In our study, the IOP of some patients began to rise at 2 months after surgery. We treated these patients antiglaucoma medications. During the whole follow-up period after modified AGV implantation, the IOP of all patients was effectively controlled, and the average IOP decreased to within the normal range. The mean (±SD) preoperative IOP was 45.48 ± 7.86 mm Hg and decreased to 14.87 ± 1.96 mm Hg at 1 week, 18.39 ± 2.25 mm Hg at 1 month, 16.61 ± 1.47 mm Hg at 3 months, and 17.48 ± 1.38 mm Hg at 6 months after modified AGV implantation. During the follow-up period, the mean postoperative IOP values differed significantly from the mean preoperative IOP (F = 256.646, *P* < .001; Table 2). The median number of antiglaucoma medications was 3 (3–4) before surgery, 0 (0–0) at 1 week after surgery, 0 (0–0) at 1 month after surgery, 0 (0–0) at 3 months after surgery, and 0 (0–1) at 6 months after surgery. The median number of antiglaucoma medications was significantly reduced from 3 (3–4) preoperatively to 0 (0–1) at the 6 months follow-up after surgery (Z = −4.248, *P* < .001).

**Table 2:** IOP before and after modified AGV implantation in NVG patients (mean ± SD).

Time	Eyes	IOP (mm Hg)
Preoperation	23	45.48 ± 7.86
1 wk postoperation	23	14.87 ± 1.96
1 mo postoperation	23	18.39 ± 2.25
3 mo postoperation	23	16.61 ± 1.47
6 mo postoperation	23	17.48 ± 1.38
*P* value		<.001
*F* value		256.646

The mean postoperative IOP was significantly lower than that before modified AGV implantation during follow-up period (F = 256.646, *P* < .001).

AGV = Ahmed glaucoma valve, IOP = intraocular pressure, NVG = neovascularization glaucoma, SD = standard deviation.

During the 6-month follow-up period, none of the following postoperative complications occurred: shallow anterior chamber, choroidal effusion, corneal endothelial decompensation, tube drainage tube-related complications (e.g., drainage tube leakage, tube erosion/exposure, and tube obstruction), fibrotic encapsulation, or endophthalmitis. However, 2 cases experienced the postoperative complication of hyphema, and 1 other patient experienced VH. The hyphema was absorbed in 1 case after conservative treatment for 1 week, and in the other patient, the hyphema was resolved after anterior chamber irrigation. Due to unsuccessful glucose management, 1 patient experienced the serious complication of VH. Subsequently, this patient underwent pars plana vitrectomy together with PRP treatment, and after these active and effective treatments, satisfactory results were obtained. According to the evaluation criteria for surgical effect, the total surgical success rate was 91% in this study. These patients were followed up for 6 to 18 months. Among 23 cases, 2 cases did not return for further consultation at 12 months, and the other 3 did not return at 18 months. Surprisingly, no drainage tube exposure occurred during the subsequent follow-up period.

## 4. Discussion

NVG typically arises secondary to a variety of retinal ischemic diseases, such as PDR, ocular ischemic syndrome, AND central retinal vein occlusion.^[3–5]^ This variety along with the complexity of both the primary disease and NVG make this condition complicated to treat and prone to poor postoperative recovery. Thus, a comprehensive treatment strategy needs to be based on the individual characteristics of NVG patients. This study included 23 patients with PDR complicated with NVG and analyzed the efficacy and safety of modified AGV implantation for the treatment of NVG. The results suggest that modified AGV implantation is a safe and effective surgical method with fewer drainage tube-related complications.

The core purpose of NVG treatment is to reduce the IOP to within the normal range, improve retina ischemia, and preserve visual function.^[3,11]^ Thus, anti-VEGF treatment is an important strategy for NVG management, as it can neutralize VEGF, leading to the regression of neovascularization within the iris and anterior chamber,^[3,11,12]^ and reduce intraoperative bleeding, thereby improving the success rate of glaucoma surgery.^[3]^ Moreover, anti-VEGF treatment could create an effective opportunity for PRP treatment. Before modified AGV implantation, all eyes in the present study received anti-VEGF treatment, and after this treatment, the regression of iris neovascularization in most participants along with an obvious reduction in intraoperative bleeding were observed. PRP treatment could be completed in the months after modified AGV implantation, as the IOP was reduced to normal levels.

Surgical options for glaucoma treatment include cyclodestructive procedures, filtering surgery, and implantation of glaucoma drainage devices.^[4]^ Filtration surgery mainly refers to trabeculectomy, but surgery failure is often caused by filtering bleb scarring.^[8]^ Extensive neovascularization on surface of the iris and anterior chamber angle can increase the risk of intraoperative bleeding.^[3]^ In addition, destruction of the blood–aqueous barrier and leakage of plasma proteins can stimulate the fibrovascular membrane and cause filtering bleb scarring, ultimately resulting in failure of the filtration surgery.^[3]^ Although cyclocryotherapy and cyclophotocoagulation are effective at decreasing IOP, they require an invasive procedure that increases the risk of ocular atrophy.^[3,11]^ Additionally, we do not recommend the surgical approach of cyclocryotherapy and cyclophotocoagulation, because it does not achieve the main goals of NVG treatment.^[3,11]^ At present, AGV implantation is the mainstay surgical method for NVG. In 1993, the AGV drainage valve was developed by Mateen Ahmed for clinical use and subsequently approved by the Food and Drug Administration.^[9,13]^ The drain valve employs the Venturi effect as its working principle: when the IOP reaches a preset threshold, the elastic membrane opens, thereby lowering the IOP to 8 to 12 mm Hg.^[13,14]^ Additionally, FP-7/FP-8 is designed with filtration holes to increase the aqueous outflow area and reduce the incidence of fibrotic encapsulation.^[13,14]^ Usually, the anterior end of the drainage tube is covered with a piece of human donor sclera, or bovine pericardial graft patch, or other suitable material, which is sutured to the sclera to prevent tube exposure.^[8,10,15]^ Alternatively, an autologous scleral flap measuring 4 mm × 6 mm and a half or 2 to 3rd thickness can be created. Then, a 22G syringe needle can be used to puncture the anterior chamber to form a short tunnel approximately 1.5 mm long.^[8,16]^ However, if the scleral flap is too thick or thin, postoperative complications such as shallow anterior chamber and low IOP are likely to occur.^[16]^ Allogeneic sclera flap use is associated with many complications, including allogeneic sclera dissolution, rejection, etc.^[16]^ Recently, Gdih et al^[17]^ investigated the safety, efficacy, and coat saving of graft-free Ahmed valve implantation through a 6 mm scleral tunnel. They conclused that the safety and efficacy of a 6mm scleral tunnel was comparable to conventional scleral-graft method.^[17]^ The difference between our method and theirs is the length of scleral tunnel and the tools used to make it. Eslami et al^[18]^ evaluated the outcome of single long scleral tunnel technique for prevevention of Ahmed valve tube exposure. The authors reported that this technique was efficacious without additional materials, and no tube exposure occurred during a relatively long follow-up period. There are subtle differences between our method and Eslami et al^[18]^ method. In their method, an 8mm and half-thick scleral tunnel is made by 60° beveled-up 2-mm crescent knife.

The highlight of the surgical method presented in this study is the modified scleral tunnel. At about 8 mm from the margin of the corneal limbus, a 22G syringe needle is used to tunnel along the arc of the eyeball to the margin of the cornea and then parallel to the iris plane into the anterior chamber, forming a scleral tunnel of about 8 mm in length between the scleral layers. This modified method avoids the need for autologous scleral flaps or use of an allogeneic scleral flap. Therefore, this surgical method has the advantages of inflicting less trauma, saving materials (human donor sclera, bovine pericardial graft patch, or others), and saving operation costs. After AGV implantation, the incidence of tube exposure ranges from 5% to 14.3%.^[8]^ Several studies have reported that tube exposure is related to the development of late endophthalmitis.^[8,19,20]^ It is speculated that the tube exposure can present a direct intraocular passage for microorganisms from the conjunctiva to the ocular surface for microorganisms.^[8,19]^ The modified scleral tunnel with a length of only 8 mm may reduce the risk of postoperative conjunctival catheter corrosion and drainage tube exposure, while also better fixing the tube and increasing the stability of the drainage tube.

Indeed, transient hypotension commonly occurs immediately after AGV implantation.^[8]^ Recent studies reported that a “hypotensive” phase was observed in 13% to 15% of eyes.^[8,21,22]^ However, the cause of the persistent and long-term hypotony after AGV implantation is not fully understood. During the surgical procedure, attention should be taken to not over-prime the tube, which might damage the valve. Perhaps damage to the valve during priming is responsible for early excessive drainage. Glaucoma experts advise that the drainage tube should be directly fixed and ligated on the sclera surface and that suturing can avoid excessive drainage in the early stage.^[23]^ However, the tightness of the suture depends on the experience of the surgeon. Sutures that are too tight or too loose will lead to poor or excessive drainage. The traditional autologous scleral flap has the disadvantages of a high incidence of leakage around the drainage tube and poor stability of the drainage tube, thus increasing the risks of shallow chamber and low IOP. In the present study, we found that modified AGV implantation for the treatment of NVG resulted in a good prognosis and few postoperative complications. During the follow-up period of 6 to 18 months, no case of drainage tube exposure was observed. Commonly, fibrotic encapsulation around the plate and long-term complications are the main causes of surgical failure.^[8]^ However, the molecular mechanism by which fibrotic encapsulation contributes to surgical failure has not yet been fully characterized. Apart from the implanted biomaterials, fibroblast proliferation and adhesion are also thought to be involved in the process of fibrotic encapsulation.^[24]^ After comprehensive treatment in the present study, no drainage tube-related complications occurred in 23 NVG cases, and the IOP of all patients was controlled within the normal range. The only complications included hyphema in 2 cases and VH in 1 case, and after effective treatment, all 3 patients achieved a satisfactory recovery. Additionally, no case of choroidal detachment occurred in our patients. The occurrence of choroidal detachment is mainly caused by a high preoperative IOP, an intraoperative sudden decrease in IOP, and a postoperative continuously low IOP. During surgery, attention should be paid to avoid a sudden decrease in IOP, and the anterior chamber can be filled with viscoelastic agent to prevent a sudden decrease in IOP. Drainage tube sutures are used to prevent excessive early drainage.

## 5. Conclusion

Long scleral tunnel technique is a safe and effective surgical method with fewer drainage tube-related complications. (e.g., drainage tube leakage and exposure). Thus, this approach can be promoted in clinical practice. Still, due to the small sample size and short follow-up duration in this study, further research with a larger sample size is needed to study the efficacy and safety of long-term modified AGV implantation in the treatment of PDR complicated with NVG.

## Author contributions

**Conceptualization:** Tao Sun.

**Data curation:** Ying Dai.

**Formal analysis:** Ying Dai, Jun-Fang Gong, Shu Zhu.

**Investigation:** Min Zhuang, Shu Zhu, Tao Sun.

**Methodology:** Ying Dai, Jun-Fang Gong.

**Project administration:** Jun-Fang Gong, Ju-Ming Zhu, Min Zhuang.

**Supervision:** Jun-Fang Gong, Min Zhuang.

**Writing – original draft:** Ying Dai.

**Writing – review & editing:** Ying Dai, Ju-Ming Zhu, Tao Sun.
